# The association between cardiorespiratory fitness, liver fat and insulin resistance in adults with or without type 2 diabetes: a cross-sectional analysis

**DOI:** 10.1186/s13102-021-00261-9

**Published:** 2021-04-16

**Authors:** Angelo Sabag, Shelley E. Keating, Kimberley L. Way, Rachelle N. Sultana, Sean M. Lanting, Stephen M. Twigg, Nathan A. Johnson

**Affiliations:** 1 NICM Health Research Institute, Western Sydney University, Westmead, NSW Australia; 2 Faculty of Medicine and Health, Discipline of Exercise and Sport Science, The University of Sydney, Camperdown, NSW Australia; 3 The Boden Collaboration for Obesity, Nutrition, Exercise and Eating Disorders, The University of Sydney, Camperdown, NSW Australia; 4 Centre for Research on Exercise, Physical Activity and Health, School of Human Movement and Nutrition Sciences, The University of Queensland, St Lucia, QLD Australia; 5 Institute for Physical Activity and Nutrition, Deakin University, Burwood, Victoria Australia; 6 Division of Cardiac Prevention and Rehabilitation, Exercise Physiology and Cardiovascular Health Lab, University of Ottawa Heart Institute, Ottawa, Canada; 7 School of Health Sciences, Faculty of Health and Medicine, University of Newcastle, Ourimbah, NSW Australia; 8 Central Clinical School, Sydney Medical School, Faculty of Medicine and Health, The University of Sydney, Camperdown, NSW Australia

**Keywords:** Obesity, Fatty liver, Exercise

## Abstract

**Background:**

Exercise-induced improvements in cardiorespiratory fitness (CRF) often coincide with improvements in insulin sensitivity and reductions in liver fat content. However, there are limited data concerning the relationship between CRF and liver fat content in adults with varying degrees of metabolic dysfunction.

**Methods:**

The aim of this study was to examine the association between CRF, liver fat content, and insulin resistance in inactive adults with obesity and with or without type 2 diabetes (T2D), via cross-sectional analysis. CRF was determined via a graded exercise test. Liver fat content was assessed via proton magnetic resonance spectroscopy and insulin resistance was assessed via homeostatic model of insulin resistance (HOMA-IR). A partial correlation analysis, controlling for age and gender, was performed to determine the association between CRF, demographic, cardiometabolic, and anthropometric variables. Independent *t* tests were performed to compare cardiometabolic outcomes between participants with T2D and participants without T2D.

**Results:**

Seventy-two adults (46% male) with a mean age of 49.28 ± 10.8 years, BMI of 34.69 ± 4.87 kg/m^2^, liver fat content of 8.37 ± 6.90%, HOMA-IR of 3.07 ± 2.33 and CRF of 21.52 ± 3.77 mL/kg/min participated in this study. CRF was inversely associated with liver fat content (*r* = − 0.28, *p* = 0.019) and HOMA-IR (*r* = − 0.40, *p* < 0.001). Participants with T2D had significantly higher liver fat content (+ 3.66%, *p* = 0.024) and HOMA-IR (+ 2.44, *p* < 0.001) than participants without T2D. Participants with T2D tended to have lower CRF than participants without T2D (− 1.5 ml/kg/min, *p* = 0.094).

**Conclusion:**

CRF was inversely associated with liver fat content and insulin resistance. Participants with T2D had lower CRF than those without T2D, however, the difference was not statistically significant. Further longitudinal studies are required to elucidate the relationship between CRF and the progression of obesity-related diseases such as T2D.

Registration: ACTRN12614001220651 (retrospectively registered on the 19th November 2014) and ACTRN12614000723684 (prospectively registered on the 8th July 2014).

**Supplementary Information:**

The online version contains supplementary material available at 10.1186/s13102-021-00261-9.

## Background

Obesity plays a significant role in the development of many chronic diseases such as hypertension, coronary heart disease, numerous cancers, and type 2 diabetes (T2D) [48]. While the relationship between obesity and increased cardiometabolic risk is well established [1], the location of adipose tissue, particularly in and/or around the liver, heart, muscles, and pancreas, also known as ectopic fat, is significantly more predictive of adverse health outcomes such as hypertension and hyperglycaemia [36]. Metabolic dysfunction-associated fatty liver disease (MAFLD), characterised by excessive liver fat (LF) content, is strongly associated with insulin resistance and is highly prevalent in individuals with T2D [41]. As T2D and MAFLD share a similar underlying pathological process, they often present alongside other disorders such as obesity, dyslipidaemia, and hypertension [49]. A known strategy for managing these risk factors is increased physical activity [23, 43], which importantly leads to improved cardiorespiratory fitness (CRF) [8]. However, individuals with T2D and/or MAFLD reportedly experience disease-related aerobic impairments, which contribute to, or are further affected by, mitochondrial dysfunction, cardiac dysfunction, insulin resistance, and diastolic dysfunction - which may manifest as low CRF [6, 44].

Low CRF is a well-established risk factor for all-cause morbidity and mortality [18] and increases an individual’s risk for developing T2D [40]. Multiple studies have shown that individuals who engage in structured exercise can improve CRF, insulin sensitivity, and reduce ectopic fat [13, 29–31], however, it is unclear whether these improvements are mediated through changes in CRF or through simply undertaking regular exercise. Recent observational data have shown that while physical activity levels were not associated with LF [16], low CRF was strongly and independently associated with MAFLD prevalence [9]. However, these findings are yet to be confirmed in participants with varying T2D-status using gold-standard LF quantification techniques and maximal exercise tests to determine CRF.

Decoupling CRF from physical activity-related improvements has proven difficult due to the interrelated nature of the two measures [24]. It could therefore be assumed that individuals who have higher physical activity and CRF levels also partake in other healthy behaviours, which provide further protection from a variety of obesity-related complications [34]. Furthermore, it is becoming increasingly accepted that both modifiable ,such as physical activity levels, and non-modifiable factors, such as gene-specific variations, contribute to variations in CRF [5, 33], with the relative genetic contribution to CRF reported to be ~ 50% [3, 33]. Consequently, it is important to explore the association between CRF and cardiometabolic risk in adults with similar levels of self-reported physical activity in order to decouple physical activity from CRF and better assess CRF-related benefits independently. There are limited studies which have investigated the relationship between CRF and insulin resistance in adults with and without T2D, and even fewer studies assessing the relationship between CRF and LF, as quantified via gold-standard proton magnetic spectroscopy (^1^H-MRS). Therefore, the aim of this cross-sectional study, was to determine the association between CRF, LF, insulin resistance, and other cardiometabolic outcomes in inactive adults with obesity, and with or without T2D. A secondary aim was to compare cardiometabolic and CRF differences between adults with T2D to those without T2D. It was hypothesised that CRF would be inversely associated with LF content and insulin resistance. It was also hypothesised that inactive adults with obesity and T2D would have significantly lower CRF than inactive adults with obesity but without T2D.

## Methods

### Participants

The participants of this study were recruited via electronic bulletins, clinical databases, and media advertisements between June 2011 and February 2019. Eligible volunteers were between the ages of 18–65 years, had a BMI ≥ 30.0 kg/m^2^, self-reported being physically inactive (exercising < 3 days/week) and/or not currently meeting physical activity guidelines [4]. All participants were screened by a medical practitioner prior to enrolment and were excluded if there was evidence of an unstable cardiac condition, uncontrolled hypertension, or uncontrolled blood glucose. The analysis included 72 volunteers from two larger intervention trials (ACTRN12614001220651 and ACTRN12614000723684) for which the results have been published elsewhere [14, 31, 46]. Eligible participants were screened via telephone interview and those who met the inclusion criteria and provided written informed consent, were enrolled in the study and were assessed at the University of Sydney (NSW, Australia). The study conformed to the ethical guidelines of the 1975 Declaration of Helsinki and the procedures were approved by the University of Sydney Human Research Ethics Committee.

### Anthropometry and blood pressure assessment

Relevant anthropometric data were collected in accordance with international standards [38]. The height of the participants was measured via stadiometer (SECA model 220 Telescopic Height Rod, Hamburg, Germany). Participants body weight was measured using Tanita BC-418 Body Composition Analyzer (Tanita Corporation, Tokyo, Japan) to the nearest 0.1 kg and BMI (kg/m^2^) was calculated. Waist circumference (WC) was measured (SECA Model 201, Hamburg, Germany) thrice horizontally, between the inferior margin of the ribs and the superior border of the iliac crest after expiration but before inspiration. After 10 to 15 mins of quiet sitting, systolic (SBP) and diastolic (DBP) blood pressures were measured manually on each arm with a sphygmomanometer (Welch Allyn^®^ 767 Series Aneroid; New York, USA) and the average of three measures was recorded from the arm which produced the highest SBP and DBP readings.

### Biochemical parameters

Venous blood was collected after an overnight fast (> 10 h) for the purpose of determining fasting blood glucose (FBG), insulin, high-sensitivity C-reactive protein (CRP), total cholesterol (TC), triglycerides (TG), high-density lipoprotein cholesterol (HDL), low-density lipoprotein cholesterol (LDL), alanine aminotransferase (ALT), and aspartate aminotransferase (AST). Concentration of plasma free fatty acids (FFA) was measured using stored plasma. All biochemical and lipid assessments were completed by a private accredited laboratory. Insulin resistance was assessed via the homeostatic model of insulin resistance (HOMA-IR) [45].

### Proton magnetic resonance spectroscopy (^1^H-MRS)

LF% was measured via ^1^H-MRS using a Phillips Intera 1.5 Telsa Achieva MRI system (Philips Medical Systems, Best, Netherlands). Spectral data were post-processed by an assessor (NAJ), who was blinded to participant details, using a magnetic resonance user interface software (jMRUI, version 5.2; www.jmrui.eu) [25, 37]. LF% ≥ 5.5% was considered consistent with the presence of metabolic dysfunction-associated fatty liver disease (MAFLD) [39]. The LF% quantification methodology employed in this study is comprehensively detailed elsewhere [31].

### Cardiorespiratory fitness

CRF was assessed via a graded maximal exercise test on an electronically-braked cycle ergometer (Lode Corival, Netherlands) under the supervision of an Accredited Exercise Physiologist [35]. All tests incorporated a three-min warm up at 35 W and 65 W for women and men, respectively, and workloads were incrementally adjusted by 25 W every 150 s until volitional fatigue as described elsewhere [15]. Heart rate, blood pressure, and ratings of perceived exertion (RPE) were obtained at each stage of exercise, with RPE measured using the Borg scale [2]. The test was terminated when the pedalling rate fell below 50 revolutions per min despite encouragement, or volitional fatigue. Peak work capacity (W_peak_) was measured [20] and peak oxygen consumption (VO_2Peak_) estimated as described previously [10]. CRF was assessed within one-week of LF assessment.

### Statistical analysis

Data were analysed using Statistical Package for the Social Sciences (SPSS version 24.0; IBM Corp., Armonk, NY, USA). All data are reported as the mean values ± standard deviation (SD) unless otherwise stated. Independent *t* tests were performed to compare differences in CRF and other biochemical and anthropometric measures between individuals with and without T2D. Homogeneity of variances was assessed via Levene’s test for equality of variance and χ^2^ for categorical data. Abnormally distributed primary outcome measures were transformed via natural logarithm prior to between-group comparisons. Partial correlation coefficients (*r*), controlling for age and sex, were used for correlations between continuous variables, and where one of the variables was dichotomous categorical data and the other was continuous. Associations between two categorical variables were assessed by χ^2^ test for independence, whereby the φ coefficient determined the magnitude of the correlation. The magnitude of correlations were qualitatively assessed as: trivial (r < 0.1), small (*r* > 0.1 to 0.3), moderate (*r* > 0.3 to 0.4), strong (*r* > 0.5 to 0.7), very strong (*r* > 0.7 to 0.9), nearly perfect (*r* > 0.9), and perfect (*r* = 1.0) [11]. Male and female participants were divided into CRF quartiles, respectively, and analysis of variance was employed to determine significant differences in cardiometabolic outcomes between CRF quartiles (lowest fitness, IQR, highest fitness). Least significant difference post-hoc comparisons were used to identify and compare significant differences between CRF quartiles. Effect sizes were calculated as standardised difference in the means and expressed as Cohen’s *d*. Statistical significance was set at *p* < 0.05. A two-tailed sensitivity analysis using the effect size of the difference in LF content between participants with T2D versus participants without T2D revealed that the study achieved 78% power (G-Power software; University of Trier, Trier, Germany).

## Results

### Participants

Participant characteristics are summarised in Table 1. A total of 72 (33 male and 39 female) volunteers participated in this study. The mean age was 49.28 ± 10.18 years, BMI 34.69 ± 4.87 kg/m^2^, WC 108.55 ± 14.43 cm, LF was 8.37 ± 6.90%, and CRF was 21.52 ± 3.77 ml/kg/min. The prevalence of MAFLD was 43% in participants without T2D , 65% in participants with T2D, and 54% amongst all participants. Participants with T2D had significantly higher WC, LF%, HOMA-IR, FBG, ALT, TC, HDL, LDL, and FFA than those without T2D (*p* < 0.05 for all). There were no differences in other variables between participants with or without T2D. Male participants had significantly higher WC, TC, HDL, LDL, CRP, HOMA-IR and SBP than female participants (Supplementary Table 1).

**Table 1:** Participant characteristics

	Normal Glucose Tolerance (*n* = 37)	Type 2 Diabetes (*n* = 35)	Total (*n* = 72)	*p*	ES (95%CI)
*Demographics and anthropometry*					
Gender (M/F)	13/24	20/15	33/39	0.610	
MAFLD (Y/N)	16/21	23/12	39/33	0.056	
Age (years)	44.84 (10.23)	53.97 (7.85)	49.28 (10.18)	**<0.001**	-1.030 (-3.93 to 1.86)
Waist Circumference (cm)	101.21 (12.30)	116.30 (12.43)	108.55 (14.43)	**<0.001**	-1.26 (-5.18 to 2.67)
BMI (kg/m^2^)	33.62 (4.52)	35.81 (5.04)	34.69 (4.87)	0.056	-0.47 (-1.99 to 1.05)
LF (%)	6.59 (5.93)	10.25 (7.43)	8.37 (6.90)	**0.008**	-0.65 (-0.84 to -0.45)
SBP (mmHg)	122.92 (14.41)	129.41 (16.85)	126.07 (15.87)	0.083	-0.43 (-5.40 to 4.55)
DBP (mmHg)	79.30 (6.99)	80.16 (9.28)	79.71 (8.14)	0.658	-0.11 (-2.72 to 2.50)
*Biochemistry*					
AST (U/L)	22.32 (9.17)	29.06 (21.05)	25.60 (16.32)	0.080	-0.43 (-5.58 to 4.73)
ALT (U/L)	22.95 (10.13)	35.17 (23.10)	28.89 (18.59)	**0.005**	-0.71 (-6.37 to 4.95)
CRP (mg/L)	4.65 (4.71)	4.11 (4.73)	4.39 (4.70)	0.628	0.12 (-1.38 to 1.62)
FBG (mmol/L)	4.28 (0.61)	8.21 (3.00)	6.19 (2.90)	**<0.001**	-1.87 (-2.56 to 1.18)
^a^Insulin (mU/L)	10.22 (5.96)	12.17 (6.63)	11.08 (6.29)	0.212	-0.32 (-2.32 to 1.68)
^a^HOMA-IR	2.00 (1.34)	4.43 (2.61)	3.07 (2.33)	**<0.001**	-1.21 (-1.86 to -0.55)
*Lipids*					
Triglycerides (mmol/L)	1.49 (0.86)	1.66 (0.67)	1.57 (0.78)	0.368	-0.23 (-0.47 to 0.02)
Total Cholesterol (mmol/L)	5.59 (1.25)	4.43 (0.80)	5.03 (1.20)	**<0.001**	1.14 (0.80 to 1.47)
HDL (mmol/L)	1.40 (0.32)	1.15 (0.26)	1.28 (0.32)	**0.001**	0.88 (0.79 to 0.98)
LDL (mmol/L)	3.53 (0.95)	2.47 (0.75)	3.02 (1.01)	**<0.001**	1.27 (1.00 to 1.55)
FFA (umol/L)	389.67 (162.24)	508.71 (198.24)	447.54 (189.09)	**0.007**	-0.68 (-58.18 to 56.83)
*Cardiorespiratory Fitness*					
VO_2peak_ (mL/kg/min)	22.24 (3.90)	20.75 (3.51)	21.52 (3.77)	0.094	0.41 (-0.77 to 1.59)

Data presented as mean (SD). *ES* effect size, *CI* confidence interval, *M* male, *F* female, *MAFLD* metabolic dysfunction-associated fatty liver disease, *Y* yes, *N* no, *BMI* body mass index, *LF%* liver fat percentage, *SBP* systolic blood pressure, *DBP* diastolic blood pressure, *AST* aspartate aminotransferase, *ALT* alanine aminotransferase, *CRP* high-sensitivity C-reactive protein, *FBG* fasting blood glucose, *HOMA-IR* homeostatic model assessment of insulin resistance, *HDL* high-density lipoprotein cholesterol, *LDL* low-density lipoprotein cholesterol, *FFA* free fatty acids, *VO*_*2peak*_ Peak oxygen consumption. ^a^HOMA-IR and insulin measures reported for 37 participants with normal glucose tolerance and 29 participants with T2D as 6 participants with T2D were undergoing exogenous insulin therapy

### Partial correlations

The associations between CRF, demographic, anthropometric, and cardiometabolic variables are summarised in Table 2. CRF was inversely associated with LF% (*r* = − 0.271), HOMA-IR (*r* = − 0.411), BMI (*r* = − 0.527), WC (*r* = − 0.490), FFA (*r* = − 0.283), CRP (*r* = − 0.344) FBG (*r* = − 0.259), insulin (*r* = − 0.403), and SBP (*r* = − 0.292) (p < 0.05 for all).

**Table 2:** Partial correlations between graded exercise test measured cardiorespiratory fitness, demographic, cardiometabolic and anthropometric variables

	1	2	3	4	5	6	7	8	9	10	11	12	13	14	15	16	17	18
**CRF (1)**																		
**LF% (2)**	−.271^*^																	
**HOMA-IR (3)**	.411^**^	−0.225																
**T2D (4)**	−0.173	0.233	−0.156															
**MAFLD (5)**	−0.184	.734^**^	−0.183	0.103														
**BMI (6)**	−.527^**^	.403^**^	−.527^**^	0.106	.387^**^													
**WC (7)**	−.490^**^	.547^**^	−.466^**^	.370^**^	.475^**^	.790^**^												
**AST (8)**	−0.199	.423^**^	−0.168	0.163	.311^**^	.268^*^	.366^**^											
**ALT (9)**	−0.231	.578^**^	−0.191	.256^*^	.391^**^	.339^**^	.466^**^	.900^**^										
**TG (10)**	−0.093	.364^**^	−0.085	0.23	0.183	0.004	0.079	.372^**^	.338^**^									
**TC (11)**	0.121	−0.078	0.132	−.492^**^	−0.032	− 0.181	−.293^*^	0.042	− 0.03	0.172								
**HDL (12)**	0.079	−0.082	0.114	−.357^**^	−0.122	− 0.065	−0.229	− 0.175	−.237^*^	−0.216	.518^**^							
**LDL (13)**	0.145	−0.162	−.433**	−.555^**^	−0.103	− 0.17	−.292^*^	−0.018	− 0.082	0.055	.945^**^	.417^**^						
**FFA (14)**	−.283^*^	.375^**^	−0.197	.317^**^	.380^**^	0.151	.286^*^	.357^**^	.351^**^	.379^**^	0.048	0.019	−0.068					
**CRP (15)**	−.344^**^	0.16	−.339^**^	0.015	0.195	.406^**^	.310^**^	.250^*^	.246^*^	0.055	−0.11	−0.074	−0.133	− 0.002				
**Glucose (16)**	−.259^*^	.268^*^	−.249^*^	.631^**^	0.159	0.108	.361^**^	0.161	0.232	.515^**^	−.245^*^	−0.185	−.416^**^	.400^**^	0.078			
**Insulin (17)**	−.403^**^	.435^**^	.835^**^	.835^**^	.411^**^	.524^**^	.534^**^	−0.008	0.214	0.242	−0.158	− 0.09	−0.263	0.177	0.18	0.177		
**SBP (18)**	−.292^*^	0.168	−.281^*^	−0.021	.332^**^	.272^*^	.395^**^	.278^*^	.287^*^	0.038	0.056	−0.103	0.023	0.233	−0.014	.238^*^	0.212	
**DBP (19)**	−0.133	0.198	−0.118	−0.114	.257^*^	0.226	.247^*^	−0.102	−0.087	0.052	0.151	.293^*^	0.031	0.067	0.017	.244^*^	0.2	.618^**^

* *p* < 0.05; ** *p* < 0.01

Data presented as correlation coefficient (*r*). *CRF* cardiorespiratory fitness, *LF%* liver fat percentage, *T2D* type 2 diabetes, *MAFLD* metabolic dysfunction-associated fatty liver disease, *BMI* body mass index, *WC* waist circumference, *AST* aspartate aminotransferase, *ALT* alanine aminotransferase, *TG* triglycerides, *TC* Total Cholesterol *HDL* high-density lipoprotein cholesterol, *LDL* low-density lipoprotein cholesterol, *FFA* free fatty acids, *CRP* high-sensitivity C-reactive protein, *SBP* systolic blood pressure, *DBP* diastolic blood pressure

### CRF and cardiometabolic outcomes

**Fig. 1:**
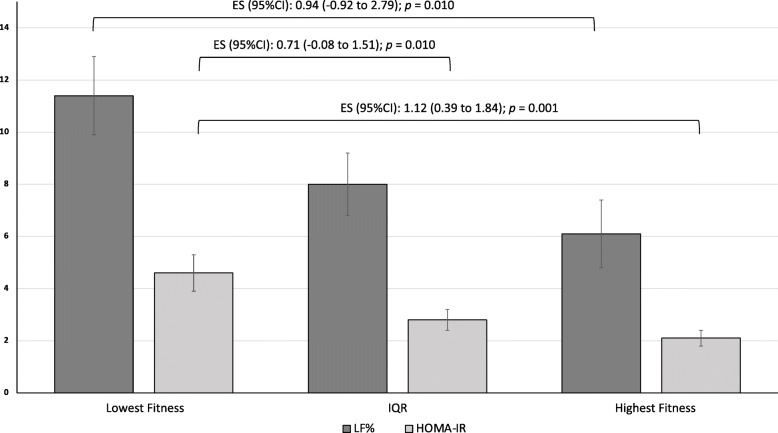
Differences in liver fat content and insulin resistance between CRF quartiles. Data presented as mean (SD). ES, effect size; CI, confidence interval; LF%, liver fat percentage; HOMA-IR, homeostatic model of insulin resistance; IQR, interquartile range. Brackets indicate significant difference

Participant characteristics stratified by CRF are summarised in Table 3. Participants with the lowest CRF had significantly higher LF% and HOMA-IR than those with the highest CRF (+ 5.31%, *p* = 0.021, + 2.50, *p* = 0.001, respectively) (Fig. 1). When compared to participants with the highest CRF, those with the lowest CRF also had significantly higher BMI (+ 7.54 kg/m^2^, *p* < 0.001), WC (+ 18.65 cm, *p* < 0.001), CRP (+ 3.80 mg/L, *p* = 0.012), FBG (+ 2.06 mmol/L, *p* = 0.034), insulin (+ 5.57 mU/L, *p* = 0.008), FFA (+ 153.34 umol/L, *p* = 0.015), and SBP (+ 16.57 mmHg, *p* = 0.001). When compared to participants with CRF scores within the interquartile range, those with the lowest CRF had significantly higher BMI (+ 7.54 kg/m^2^, *p* < 0.001), WC (+ 18.65 cm, *p* < 0.001), CRP (+ 3.93 mg/L, *p* = 0.003) and SBP (+ 10.89 mmHg, *p* = 0.014).

**Table 3:** Participant characteristics when stratified by cardiorespiratory fitness

	Lowest Fitness, *n* = 18 (16.71 ml/kg/min)	IQR, *n* = 36 (21.49 ml/kg/min)	Highest Fitness, *n* = 18 (26.36 ml/kg/min)	Between group *p*	ES (95%CI)
*Demographics and anthropometry*					
Gender (M/F)	8/10	17/19	8/10	0.974	
Type 2 Diabetes (Y/N)	11/7	18/18	6/12	0.250	
MAFLD (Y/N)	15/3	16/20	8/10	**0.015**^**b, c**^	
Age (years)	54.56 (9.94)	47.33 (9.48)	47.89 (10.47)	**0.037**^**b, c**^	0.67 (-2.57 to 3.91)
Waist Circumference (cm)	119.03 (15.38)	107.39 (12.64)	100.38 (10.70)	**<0.001**^**b, c**^	1.45 (-2.76 to 5.65)
BMI (kg/m^2^)	39.81 (4.29)	33.34 (3.55)	32.27 (4.12)	**<0.001**^**b, c**^	1.85 (0.51 to 3.18)
LF (%)	11.40 (6.25)	8.00 (7.48)	6.08 (5.40)	**0.033**^**b**^	0.94 (-0.92 to 2.79)
SBP (mmHg)	135.66 (20.17)	124.77 (13.08)	119.09 (11.86)	**0.004**^**b, c**^	1.03 (-4.22 to 6.28)
DBP (mmHg)	83.21 (11.33)	78.60 (6.58)	78.44 (6.46)	0.107 ^**b**^	0.53 (-2.40 to 3.46)
*Biochemistry*					
AST (U/L)	30.39 (22.57)	25.78 (16.16)	20.44 (3.62)	0.189	0.633 (-4.50 to 5.77)
ALT (U/L)	34.72 (22.72)	29.22 (18.81)	22.39 (10.82)	0.136 ^**b**^	0.71 (-4.94 to 6.36)
CRP (mg/L)	7.30 (6.72)	3.38 (3.41)	3.51 (3.30)	**0.008**^**b, c**^	0.74 (-0.94 to 2.42)
FBG (mmol/L)	7.14 (3.16)	6.26 (3.14)	5.01 (1.59)	0.101 ^**b**^	0.88 (0.08 to 1.67)
^a^Insulin (mU/L)	14.29 (7.79)	10.68 (6.16)	8.72 (3.37)	**0.026**^**b**^	0.96 (-0.95 to 2.86)
^a^HOMA-IR	4.56 (2.89)	2.82 (2.06)	2.08 (1.44)	**0.004**^**b, c**^	1.12 (0.39 to 1.84)
*Lipids*					
Triglycerides (mmol/L)	1.61 (0.75)	1.57 (0.73)	1.54 (0.91)	0.973	0.09 (-0.18 to 0.35)
Total Cholesterol (mmol/L)	4.93 (1.34)	4.93 (1.24)	5.30 (0.95)	0.527	-0.33 (-0.70 to 0.04)
HDL (mmol/L)	1.30 (0.30)	1.22 (0.34)	1.38 (0.29)	0.223	-0.28 (-0.37 to -0.19)
LDL (mmol/L)	2.88 (1.10)	2.96 (1.03)	3.27 (0.87)	0.464	-0.41 (-0.72 to -0.09)
FFA (umol/L)	531.83 (196.93)	439.92 (188.65)	378.49 (156.79)	**0.047**^b^	0.89(-55.62 to 57.39)

Data presented as mean (SD). *IQR* interquartile range, *ES* effect size, *CI* confidence interval, *M* male, *F* female, *MAFLD* metabolic dysfunction-associated fatty liver disease, *Y* yes, *N* no, *BMI* body mass index, *LF%* liver fat percentage, *SBP* systolic blood pressure, *DBP* diastolic blood pressure, *AST* aspartate aminotransferase, *ALT* alanine aminotransferase, *CRP* high-sensitivity C-reactive protein, *FBG* fasting blood glucose, *HOMA-IR* homeostatic model assessment of insulin resistance, *HDL* high-density lipoprotein cholesterol, *LDL* low-density lipoprotein cholesterol, *FFA* free fatty acids, *VO*_*2*_ Peak oxygen consumption. ^a^HOMA-IR and insulin measures reported for 37 participants with normal glucose tolerance and 29 participants with T2D as 6 participants with T2D were undergoing exogenous insulin therapy. ^b^significant difference between lowest and highest fitness; ^c^significant difference between lowest fitness and IQR

## Discussion

This study is one of the first to show significant associations between key cardiometabolic risk factors, such as LF content, quantified via gold-standard ^1^H-MRS, and CRF in individuals with and without T2D. The analyses showed that the prevalence of MAFLD in participants with T2D was 65%, which is consistent with previous reports [50]. When compared to participants without T2D, those with T2D had significantly higher cardiometabolic abnormalities. Furthermore, adults with T2D also had lower CRF than those without T2D, however, the difference was not statistically significant. Further analyses showed that even relatively small variations in CRF were associated with increased LF content and insulin resistance, which were significantly higher in individuals with relatively low CRF than those with relatively high CRF. Similarly, individuals with the highest CRF had lower cardiometabolic and inflammatory abnormalities than those with the lowest CRF.

### The association between cardiorespiratory fitness and liver fat

In accordance with the original hypothesis, CRF was inversely associated with LF content. This finding supports previously reported data, where Kantartzis and colleagues showed that LF content was inversely associated with CRF (*r* = − 0.22, *p* = 0.005) in a mixed sample of adults with MAFLD or adults at risk of metabolic disease [12]. Similarly, the findings from The Young Finns study showed that for every 1 ml/kg/min increase in CRF, the risk of MAFLD significantly declined (risk ratio = 0.90, 95% confidence interval 0.88 to 0.93; *p* < 0.001), however, LF content was measured up to three years after initial assessment of CRF and done so using ultrasound, which is less accurate than ^1^H-MRS [27]. Another study reported low CRF was inversely associated with increasing MAFLD activity and steatohepatitis severity measured via liver biopsy and sampled up to four months after CRF assessment [19]. The results of the study reported herein add to previous findings and suggest that even relatively small variations in CRF are associated with increased LF in inactive adults with or at risk of T2D. Importantly, the assessments of CRF and LF content were undertaken within a narrow timeframe (< 1 week) and LF% was quantified using gold-standard ^1^H-MRS, thus highlighting the novelty and methodological rigour of the present study.

### The association between cardiorespiratory fitness and insulin resistance and other cardiometabolic outcomes

In accordance with the original hypothesis, CRF was inversely associated with insulin resistance. Further analyses revealed that, on average, individuals with relatively poor fitness had abnormally high levels of insulin resistance, blood pressure, inflammation and LF content, whereas individuals with relatively high fitness, only had slightly abnormal LF content (11.40% vs. 6.08% for low vs. high CRF, respectively). These data, which are supported by previous findings [12, 13, 22, 29–31, 47], suggest that CRF may play an important role in the context of metabolic disease.

A recent meta-analysis reported that CRF was inversely associated with T2D prevalence in a dose-dependent manner [28]. The results of the current study showed that while participants with T2D had lower CRF than those without T2D, the difference was not statistically significant. Furthermore, as physical activity has been shown to be inversely associated with LF content independent of BMI [17], only inactive participants were included in this study in an attempt to control for higher levels of physical activity - which incur cardiometabolic benefits. Because of this, the mean level of CRF of participants was quite low at 21.5 mL/kg/min. Reports show that CRF < 29.1 mL/kg/min increases the likelihood of developing metabolic syndrome six-fold [21], consequently a greater number of participants with higher levels of CRF are required to provide more robust results.

### Mechanisms

While the mechanistic interplay between low CRF, MAFLD, and T2D remains unclear, it is purported that low CRF and ensuing mitochondrial defects contribute to the incomplete oxidation of fatty acids, which contribute to the accumulation of fatty acid by-products, such as ceramides and diacylglycerol in skeletal muscle and liver cells. The intracellular accumulation of these by-products impair insulin signalling pathways and contribute to insulin resistance [7, 26]. Importantly, T2D-related exercise intolerance appears to be reversed by regular exercise, which is made evident by the amelioration of skeletal muscle mitochondrial impairments, as well as improved insulin sensitivity, and CRF [29, 32, 42].

### Strengths

This study adds to existing literature by highlighting the importance of CRF for metabolic health in adults with or at risk of metabolic disease. While previous studies have shown similar findings [12, 27], this is the first study to do so involving a mixed sample of inactive adults with obesity and with or without T2D. Furthermore, CRF and LF were measured within a week of each other, whereas previous studies had measured CRF up to three years after LF assessment [27]. Finally, this study quantified LF% using ^1^H-MRS, which is currently considered the gold-standard non-invasive technique for LF quantification.

### Limitations

This study has limitations that should be considered when interpreting the results. Firstly, the results of this study, by nature, incorporated measures of CRF and LF content at a time-specific point and did not track the progression of any outcome to determine their relative importance in the development of MAFLD or T2D disease progression. Secondly, this study was completed using baseline data from previous interventional studies which prevented an a priori power analysis. However, the two-tailed sensitivity analysis revealed that the study achieved 78% power, which is just shy of the commonly accepted 80% value. Thirdly, this study assessed the amount of LF% per se and the methodology employed cannot determine the amount of fibrosis or classification of more severe liver diseases such as non-alcoholic steatohepatitis and/or their association with CRF. Additionally, while ^1^H-MRS is currently considered the gold-standard non-invasive measurement technique for LF%, HOMA-IR is comparatively more limited and cannot provide inference into tissue-specific impairments in insulin sensitivity. Fourthly, although CRF was assessed using a validated graded exercise test model [10], the gold-standard of aerobic capacity testing involves direct measures of gas analysis, and where possible, this method should be implemented. Finally, while an attempt was made to control for high levels of physical activity by only recruiting individuals who reported to be inactive (exercising < 3 days/week), inter-participant variations in physical activity levels likely contributed to the associations between CRF and LF.

## Conclusions

The results of this study showed that CRF was inversely associated with ^1^H-MRS-quantified LF content in inactive adults with obesity. CRF was also inversely associated with insulin resistance and other key cardiometabolic risk factors. Adults with T2D had lower CRF than adults without T2D, however, the difference did not achieve statistical significance. These findings suggest that CRF may play a key role in metabolic dysfunction, however, further longitudinal studies are required to elucidate the relationship between CRF and the progression of obesity-related diseases such as MAFLD and T2D.

## Supplementary Information

**Additional file 1: Supplementary Table 1**: Participant characteristics stratified by gender.

## Data Availability

The datasets used and/or analysed during the current study are available from the corresponding author on reasonable request.

## Abbreviations

CRF: Cardiorespiratory fitness
LF: Liver fat
^1^H-MRS: Proton magnetic resonance spectroscopy
HOMA-IR: Homeostatic model assessment of insulin resistance
T2D: Type 2 diabetes
MAFLD: Metabolic dysfunction-associated fatty liver disease
WC: Waist circumference
SBP: Systolic blood pressure
DBP: Diastolic blood pressure
FBG: Fasting blood glucose
CRP: C-reactive protein
TC: Total cholesterol
TG: Total triglyceride
HDL: High density lipoprotein cholesterol
LDL: Low-density lipoprotein cholesterol
ALT: Alanine aminotransferase
AST: Aspartate aminotransferase
RPE: Rate of perceived exertion
W_peak_: Peak work capacity
VO_2Peak_: Peak oxygen capacity
