# Supplementary material for: The association between cardiorespiratory fitness, liver fat and insulin resistance in adults with or without type 2 diabetes: a cross-sectional analysis
Source: BMC Sports Sci Med Rehabil. 2021 Apr 16;13:40. doi: 10.1186/s13102-021-00261-9 (PMC8050897; doi:10.1186/s13102-021-00261-9)
Supplement: Supplementary file 1 — Additional file 1: Supplementary Table 1: Participant characteristics stratified by gender. [file 13102_2021_261_MOESM1_ESM.docx]

**Supplementary Table 1: Participant characteristics stratified by gender.**

|  | Male (*n* =33) | Female (*n* =39) | *p* |
| --- | --- | --- | --- |
| *Demographics and anthropometry* |  |  |  |
| T2D (Y/N) | 20/13 | 15/24 | 0.063 |
| MAFLD (Y/N) | 21/12 | 21/18 | 0.141 |
| Age (years) | 48.88 (11.97) | 49.62 (8.53) | 0.769 |
| Waist Circumference (cm) | 116.70 (12.29) | 101.64 (12.47) | **<0.001** |
| BMI (kg/m^2^) | 35.69 (5.35) | 33.84 (4.31) | 0.115 |
| LF% | 8.90 (6.46) | 7.92 (7.31) | 0.550 |
| SBP (mmHg) | 130.87 (16.59) | 122.01 (14.21) | **0.019** |
| DBP (mmHg) | 81.40 (8.73) | 78.29 (7.40) | 0.112 |
| *Biochemistry* |  |  |  |
| AST (U/L) | 28.30 (15.44) | 23.31 (16.89) | 0.194 |
| ALT (U/L) | 32.27 (14.46) | 26.03 (21.24) | 0.144 |
| CRP (mg/L) | 3.13 (2.71) | 5.46 (5.70) | **0.027** |
| FBG (mmol/L) | 6.90 (3.32) | 5.59 (2.37) | 0.063 |
| ^Insulin (mU/L) | 12.52 (7.01) | 9.80 (5.36) | 0.085 |
| ^HOMA-IR | 3.73 (2.63) | 2.48 (1.87) | **0.031** |
| *Lipids* |  |  |  |
| Triglycerides (mmol/L) | 2.85 (6.90) | 1.5 (0.70) | 0.271 |
| Total Cholesterol (mmol/L) | 4.50 (0.90) | 5.47 (1.24) | **<0.001** |
| HDL (mmol/L) | 1.10 (0.27) | 1.43 (0.29) | **<0.001** |
| LDL (mmol/L) | 2.62 (0.87) | 3.35 (1.00) | **0.002** |
| FFA (umol/L) | 416.09 (177.33) | 474.15 (196.83) | 0.192 |
| *Cardiorespiratory Fitness* |  |  |  |
| VO_2peak_ (mL/kg/min) | 21.87 (3.72) | 21.22 (3.51) | 0.469 |

M: male; F: female; MAFLD: metabolic dysfunction-associated fatty liver disease; Y: yes; N: no; BMI: body mass index; LF%: liver fat percentage; SBP: systolic blood pressure; DBP: diastolic blood pressure; AST: aspartate aminotransferase; ALT: alanine aminotransferase; CRP: high-sensitivity C-reactive protein; FBG: fasting blood glucose; HOMA-IR: homeostatic model assessment of insulin resistance; HDL: high-density lipoprotein cholesterol; LDL: low-density lipoprotein cholesterol; FFA: free fatty acids; VO_2peak_: Peak oxygen consumption. ^HOMA-IR and insulin measures reported for 31 Male participants and 35 female participants as 6 participants with T2D were undergoing exogenous insulin therapy.
